# Development of an Indirect ELISA Based on the PEDV S Protein and Evaluation of Its Correlation With Neutralizing Antibody and Immune Protection

**DOI:** 10.1155/tbed/6185969

**Published:** 2025-12-28

**Authors:** Guangming Ma, Guangli Hu, Guangcai Ren, Wang Ma, Shuqiong Zhang, Hanqin Shen, Yuanjia Liu

**Affiliations:** ^1^ College of Coastal Agricultural Sciences, Guangdong Ocean University, Zhanjiang, 524088, China, gdou.edu.cn; ^2^ Key Laboratory of Manufacture Technology of Veterinary Bioproducts, Ministry of Agriculture and Rural Affairs, Zhaoqing Dahuanong Biology Medicine Co., Ltd, Zhaoqing, Guangdong, 526200, China, agri.gov.cn; ^3^ State Key Laboratory of Biocontrol, Guangzhou Higher Education Mega Center, School of Life Sciences, Sun Yat-sen University, Guangzhou, 510000, China, sysu.edu.cn; ^4^ Canton Biologics Co. Ltd, Guangzhou, 510700, China; ^5^ Neimenggu Chia Tai Food Co. Ltd, Huhehaote, 010000, China

## Abstract

Porcine epidemic diarrhea virus (PEDV) is an enteric coronavirus that infects pigs of all ages. Notably, neonatal piglets (≤7 days old) in naïve herds can experience case fatality rates approaching 100%, with hallmark clinical signs of vomiting, diarrhea, and dehydration, thereby representing a major threat to swine herd health. Serum PEDV‐specific IgG serves as a key serological marker of vaccine‐induced herd immunity. Accordingly, we developed an indirect ELISA (iELISA) using Chinese hamster ovary (CHO) cell‐expressed recombinant PEDV spike (S) protein as the coating antigen. Checkerboard titration identified the optimal antigen‐coating concentration and serum dilution as 1 μg/mL and 1:300, respectively. Based on sample‐to‐positive (S/P) ratios from 50 PEDV‐negative sera, the diagnostic cutoff was set at 0.796, corresponding to the mean plus 3 standard deviation (SD) of the negative control group (*n* = 50). The intra‐ and interassay coefficients of variation (CVs) were both <6.30%, indicating good precision (repeatability) and reproducibility. Specificity testing showed no detectable cross‐reactivity with sera positive for five common porcine viruses (porcine deltacoronavirus (PDCoV), transmissible gastroenteritis virus (TGEV), porcine group A rotavirus (PoRVA), porcine reproductive and respiratory syndrome virus (PRRSV), and African swine fever virus (ASFV)). Moreover, PEDV‐specific IgG measured by iELISA correlated strongly with virus‐neutralizing (VN) antibody titers (*r* = 0.95), and both metrics were associated with protective outcomes at the herd level. Collectively, this assay provides an effective tool for evaluating protective immunity following PEDV vaccination.

## 1. Introduction

Porcine epidemic diarrhea (PED) is caused by PED virus (PEDV), a coronavirus that infects pigs of all ages and primarily targets intestinal epithelial cells [1]. Piglets within the first 7 days of life experience the most severe disease, characterized by vomiting, watery diarrhea, and dehydration, and can exhibit mortality rates approaching 100%, resulting in a profound worldwide economic impact on pig production [2]. Classified within the genus *Alphacoronavirus*, PEDV is a +ssRNA (positive‐sense single‐stranded RNA) virus; electron microscopy reveals spherical virions with coronate (crown‐like) spikes, measuring roughly 95–190 nm in diameter and harboring an ~28‐kb genome [3]. By genotype, PEDV strains are categorized as G1 (classical) and G2 (variant). G1 strains typically exhibit milder pathogenicity, whereas G2 variants are associated with higher mortality [4–6]. The earliest report of PEDV in swine herds dates to 1971 in the United Kingdom, and in 1978 the agent was isolated from diarrheic piglets and named the CV777 strain [7]. Thereafter, PEDV became prevalent in Europe. During the late 1970s and 1980s, Belgium, France, Germany, and other countries experienced outbreaks marked by profuse diarrhea, vomiting, and severe dehydration in infected herds [8]. Since 2010, PEDV epidemiology has shifted substantially. A highly virulent G2 variant first identified in Guangdong, China, spread rapidly throughout the country and reached the United States in 2013 [8, 9], where it precipitated nationwide epidemics with millions of piglet fatalities and devastating economic consequences [10].

From 5′ to 3′, the PEDV genome comprises the 5′ untranslated region (UTR), ORF1a, ORF1b, S, ORF3, E, M, and N, followed by the 3′ UTR [6, 11, 12]. The genome harbors multiple open reading frames (ORFs) encoding structural proteins (SPs) and nonstructural proteins (NSPs) that are essential for viral replication, transcription, assembly, and immune evasion [13]. ORF1a and ORF1b occupy the 5′‐proximal region of the genome. ORF1a encodes the polyprotein pp1a, which is processed by viral proteases into 11 NSPs (NSP1–NSP11). Together with ORF1a, ORF1b encodes the polyprotein pp1ab, which is cleaved to generate NSP12–NSP16 [6, 11]. The major SPs at the 3′ end include spike (S), nucleocapsid (N), membrane (M), and envelope (E), which mediate viral entry, assembly, and dissemination [12]. The S protein is a transmembrane glycoprotein of ~180 kDa composed of two subunits, S1 and S2: S1 contains the receptor‐binding domain (RBD) that recognizes host cell receptors, whereas S2 mediates fusion of the viral envelope with the host cell membrane to facilitate entry [14, 15]. The S protein is the principal immunogen of PEDV, eliciting robust virus‐specific host responses, and thus serves as an ideal antigen for serological assay development [16–18].

Well‐structured immunization regimens elicit robust serum IgG responses in sows [19–21]. These antibodies protect sows against infection and are also passively transferred via colostrum to piglets, providing critical early‐life protection [22–24]. Routine serological surveillance of sow herds—tracking PEDV‐specific IgG seropositivity, mean titers (sample‐to‐positive [S/P] ratios), and temporal trends—enables evaluation of vaccine effectiveness, prediction of piglet protection rates, and optimization of vaccination timing [16, 25]. In this study, we developed an indirect ELISA (iELISA) using a Chinese hamster ovary (CHO)‐expressed PEDV CH/HNDZ/2022 strain spike protein as the coating antigen to quantify PEDV‐specific IgG in swine sera and to assess vaccine‐induced immunity associated with protection. Collectively, the S protein‐based IgG iELISA demonstrated robust analytical performance and practical utility for herd‐level surveillance.

## 2. Results and Discussion

The spike (S) protein was recombinantly expressed using a CHO cell expression system from the PEDV CH/HNDZ/2022 strain (GIIc genotype). By checkerboard titration, we determined that an S protein coating concentration of 1 µg/mL, a primary serum dilution of 1:300, and a secondary antibody dilution of 1:60,000 yielded the highest positive‐to‐negative (P/N) ratio (Table 1). For blocking, 2% bovine serum albumin (BSA) solution for 180 min achieved the best signal‐to‐noise ratio (Figure 1A). Regarding incubation times, a 30 min primary antibody incubation (Figure 1B), a 60 min secondary antibody incubation (Figure 1C), and a 15 min substrate incubation were identified as optimal (Figure 1D). We assayed 50 PEDV‐seronegative and 30 PEDV‐seropositive serum samples, which yielded a mean S/P ratio of 0.385 with a standard deviation (SD) of 0.0283 for the negative controls. Accordingly, the iELISA cutoff was set at 0.796, calculated as the mean plus 3 SD. Serum samples with optical density [OD]_450_ ≥ 0.796 were classified as positive, whereas those with OD_450_ < 0.796 were classified as negative (Figure 2A). To assess specificity, we tested five heterologous positive sera in duplicate using the PEDV IgG iELISA. These included sera positive for porcine reproductive and respiratory syndrome virus (PRRSV), transmissible gastroenteritis virus (TGEV), porcine group A rotavirus (PoRVA), porcine deltacoronavirus (PDCoV), and African swine fever virus (ASFV). The S/P ratio was defined as S/P = (OD_sample_ − OD_negative control_)/(OD_positive control_ − OD_negative control_). In this experiment, the positive control OD values were 1.012 and 1.008 (mean 1.010), and the negative control OD values were 0.072 and 0.069 (mean 0.0705), giving a positive‐minus‐negative (PC − NC) difference of 0.9395, which satisfied the predefined quality control criteria. The five heterologous sera yielded OD_450_ readings from 0.064 to 0.201, corresponding S/P values from −0.01 to 0.14—all well below the cutoff. All samples were correctly classified as negative, with no cross‐reactivity observed in the PEDV IgG iELISA. Duplicate measurements were highly concordant, with *Δ*S/P ≤ 0.01 for all samples. Notably, even for TGEV and PDCoV—both coronaviruses related to PEDV—their positive sera yielded S/P values near the negative control level, further supporting the high specificity of the kit (Figure 2B).

Figure 1Optimization of iELISA conditions. (A) Optimal blocking solution and blocking time. (B) Optimal serum incubation time. (C) Optimal secondary antibody incubation time. (D) Optimal substrate–enzyme reaction time.

**Figure:**
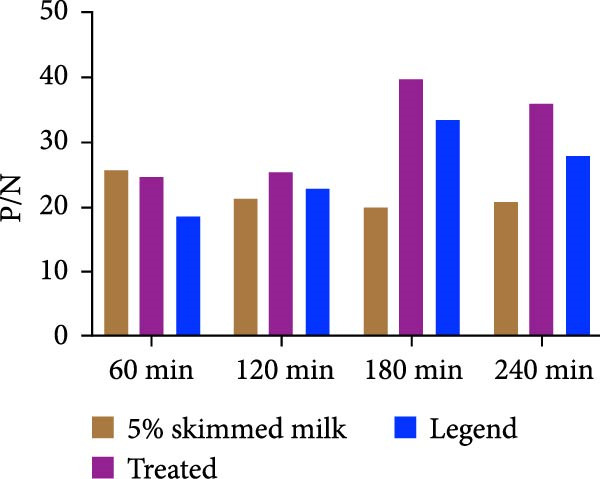
(A)

**Figure:**
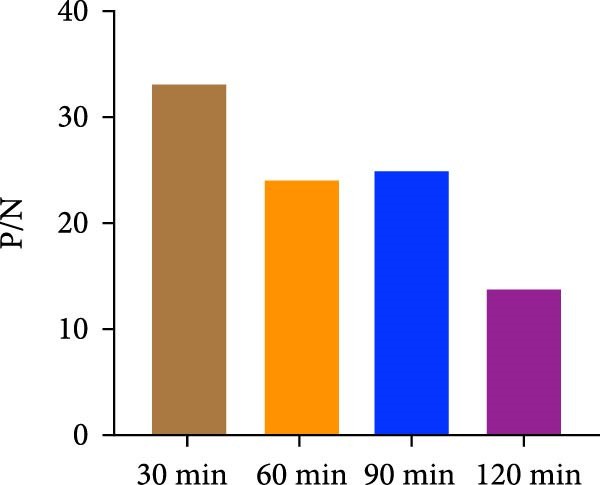
(B)

**Figure:**
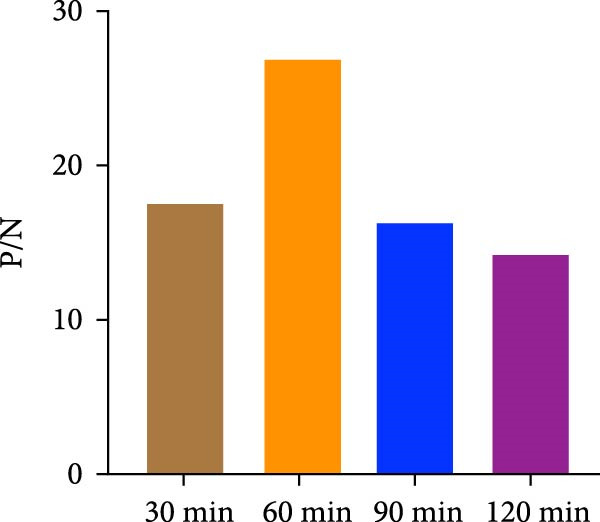
(C)

**Figure:**
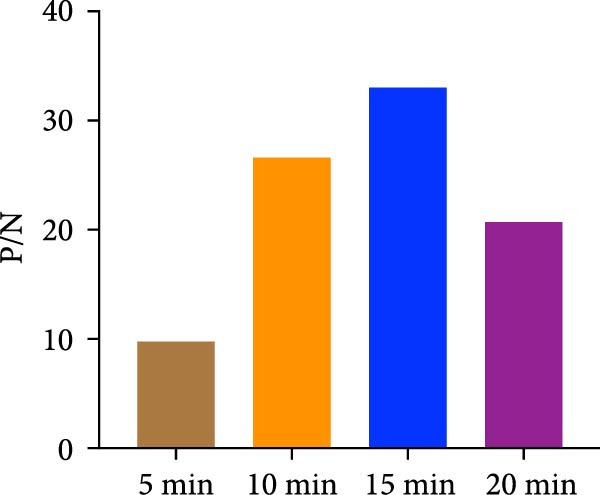
(D)

Figure 2Evaluation and application of the established iELISA. (A) Determination of the cutoff value using PEDV‐negative and PEDV‐positive sera. (B) Analytical specificity of the iELISA assessed with antisera against porcine pathogens, including PDCoV, TGEV, PoRVA, PRRSV, and ASFV.

**Figure:**
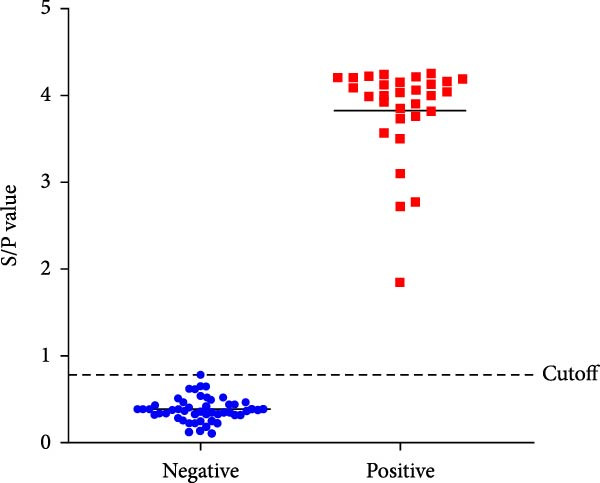
(A)

**Figure:**
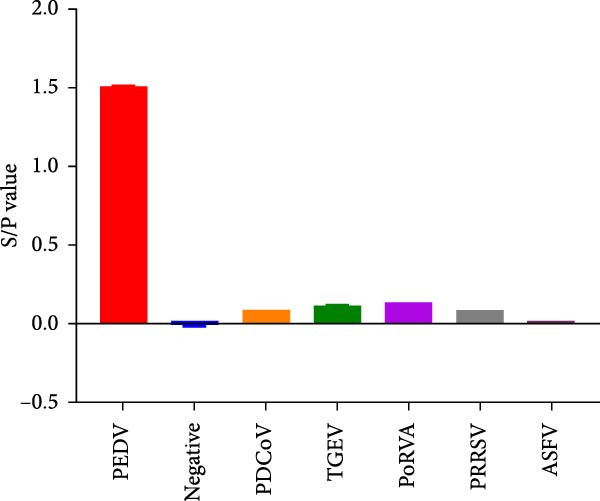
(B)

**Table 1:** Optimized iELISA parameters for antigen coating, serum dilution, and secondary antibody dilution.

Antige (µg/mL)	Serum dilution	Secondary antibody dilution	Serum dilution			
			1:100	1:200	1:300	1:400
0.5	Negative	10,000	0.167	0.067	0.067	0.058
	Positive	10,000	2.452	2.112	1.899	1.555
	P/N	10,000	14.671	31.493	28.209	26.724

0.5	Negative	30,000	0.152	0.066	0.066	0.062
	Positive	30,000	2.223	1.723	1.542	1.212
	P/N	30,000	14.605	26.061	23.333	19.516

0.5	Negative	60,000	0.152	0.071	0.071	0.058
	Positive	60,000	1.572	1.488	1.212	0.876
	P/N	60,000	10.329	20.845	17.042	15.000

1.0	Negative	10,000	0.184	0.098	0.089	0.077
	Positive	10,000	4.044	2.889	2.589	1.987
	P/N	10,000	21.957	29.388	28.989	25.714

1.0	Negative	30,000	0.152	0.089	0.076	0.068
	Positive	30,000	3.723	2.812	2.434	1.433
	P/N	30,000	24.474	31.573	31.974	21.029

1.0	Negative	60,000	0.118	0.071	0.054	0.065
	Positive	60,000	2.557	2.443	2.413	1.212
	P/N	60,000	21.610	34.366	44.630	18.615

1.5	Negative	10,000	0.218	0.207	0.141	0.071
	Positive	10,000	4.214	3.878	2.614	1.988
	P/N	10,000	19.312	18.696	18.511	27.887

1.5	Negative	30,000	0.207	0.156	0.152	0.066
	Positive	30,000	3.852	2.977	2.587	1.887
	P/N	30,000	18.599	19.038	16.974	28.485

1.5	Negative	60,000	0.198	0.137	0.144	0.075
	Positive	60,000	3.413	2.567	2.442	1.765
	P/N	60,000	17.222	18.686	16.944	23.467

2.0	Negative	10,000	0.334	0.318	0.304	0.268
	Positive	10,000	4.212	4.075	3.898	3.655
	P/N	10,000	12.605	12.799	12.796	13.619

2.0	Negative	30,000	0.283	0.255	0.221	0.222
	Positive	30,000	3.854	3.785	3.513	3.076
	P/N	30,000	13.604	14.824	15.882	13.829

2.0	Negative	60,000	0.227	0.221	0.208	0.198
	Positive	60,000	3.417	3.044	3.042	2.487
	P/N	60,000	15.022	13.818	14.615	12.525

To evaluate interassay precision, 36 serum samples were each tested once across three independently produced assay lots, and the mean, SD, and coefficient of variation (CV%) were calculated as CV (%) = (SD/mean) × 100 (Table 2). Overall, the CV values ranged from 1.2% to 6.3%, with a median of 3.18% (interquartile range = 2.21%–3.81%) and a mean of 3.16%. Notably, 33 of 36 samples (91.7%) exhibited CV ≤ 5%, and all samples had CV < 10%, demonstrating high between‐lot reproducibility and assay precision. As shown in Table 3, we assessed the analytical sensitivity of our in‐house iELISA in comparison with a commercial PEDV IgG ELISA kit (Guangdong Biaoyun Biotechnology Co., Ltd., China) by evaluating endpoint dilution titers across eight serum samples. The results indicated that for most samples (1, 2, 3, 4, 6, and 8), endpoint titers were consistent between the two assays. Notably, samples 5 and 7 exhibited higher endpoint titers in our iELISA (1:4800 and 1:2400) than in the commercial kit (1:2400 and 1:1200), suggesting greater analytical sensitivity of the developed assay for these specimens. Overall, the iELISA demonstrated high concordance in analytical sensitivity with the commercial kit, outperforming it in a subset of samples.

**Table 2:** Evaluation of iELISA reproducibility (intra‐ and interassay variability).

Sample	OD			Mean OD	SD	CV%
	Lot 1	Lot 2	Lot 3			
1	2.78	2.77	2.87	2.807	0.055	2.00
2	2.79	2.72	2.87	2.793	0.075	2.70
3	2.93	2.83	2.89	2.883	0.05	1.70
4	2.95	3	3.04	2.997	0.045	1.50
5	2.73	2.9	3.05	2.893	0.16	5.50
6	2.32	2.37	2.4	2.363	0.04	1.70
7	2.87	2.83	3.03	2.91	0.106	3.60
8	3.05	3.12	3.21	3.127	0.08	2.60
9	2.05	2.13	2.19	2.123	0.07	3.30
10	1.98	2.13	2.12	2.077	0.084	4.00
11	2.7	2.84	2.87	2.803	0.091	3.20
12	0.487	0.512	0.506	0.502	0.013	2.60
13	2.56	2.6	2.67	2.61	0.056	2.10
14	2.7	2.69	2.79	2.727	0.055	2.00
15	2.29	2.34	2.42	2.35	0.066	2.80
16	2.44	2.57	2.64	2.55	0.101	4.00
17	2.44	2.48	2.55	2.49	0.056	2.20
18	1.49	1.61	1.61	1.57	0.069	4.40
19	2.64	2.59	2.49	2.573	0.076	3.00
20	2.81	2.85	2.67	2.777	0.095	3.40
21	2.53	2.51	2.47	2.503	0.031	1.20
22	2.3	2.43	2.28	2.337	0.081	3.50
23	2.87	3.03	2.85	2.917	0.099	3.40
24	1.92	2.01	1.96	1.963	0.045	2.30
25	0.333	0.338	0.326	0.332	0.006	1.80
26	0.462	0.518	0.491	0.49	0.028	5.70
27	0.348	0.392	0.358	0.366	0.023	6.30
28	0.919	0.994	0.94	0.951	0.039	4.10
29	2.63	2.65	2.55	2.61	0.053	2.00
30	0.812	0.873	0.858	0.848	0.032	3.70
31	1.08	1.15	1.12	1.117	0.035	3.10
32	0.603	0.63	0.607	0.613	0.015	2.40
33	2.46	2.61	2.61	2.56	0.087	3.40
34	1.88	2.05	1.89	1.94	0.095	4.90
35	2.85	3.07	2.86	2.927	0.124	4.20
36	3.02	3.22	3.13	3.123	0.1	3.20

**Table 3:** Comparative sensitivity of the iELISA and a commercial iELISA kit.

Sample ID	Sample type	Endpoint dilution (titer)	
		In‐house PEDV IgG iELISA	Commercial PEDV IgG ELISA kit
1	Serum	1:1200	1:1200
2	Serum	1:1200	1:1200
3	Serum	1:1200	1:1200
4	Serum	1:2400	1:2400
5	Serum	1:4800	1:2400
6	Serum	1:600	1:600
7	Serum	1:2400	1:1200
8	Serum	1:4800	1:2400

A total of 189 porcine serum samples were tested in parallel using a commercial PEDV IgG ELISA kit (Guangdong Biaoyun Biotechnology Co., Ltd., China) and the assay developed in this study. Relative to the commercial kit, our assay showed two discordant positives and nine discordant negatives, yielding an overall agreement of 94.18% (Table 4). Most discrepancies occurred among borderline (near‐cutoff) specimens, further supporting high concordance between the two assays in positive/negative classification.

**Table 4:** Clinical agreement of serum results between the commercial iELISA kit and the in‐house iELISA.

Parameters	In‐house PEDV IgG iELISA	Commercial PEDV IgG ELISA kit
Number of discordant positive samples	2	/
Number of discordant negative samples	9	
Total discordant samples	11	
Overall agreement (%)	94.18	
Total number of samples	189	

*Note:* The commercial ELISA kit was used as the comparator.

Following the established protocol, an inactivated vaccine based on the PEDV CH/HNDZ/2022 strain was prepared, and 4‐week‐old piglets were immunized on days 0 and 14. IgG titers peaked 2–3 weeks after the second immunization, then declined gradually and persisted for at least 12 weeks (Figure 3A). Neutralizing antibodies were subsequently measured and correlated with IgG, revealing a strong association (*r* = 0.95); Figure 3B). Correlation coefficients were calculated using Pearson correlation, with *p* < 0.05 considered significant.

Figure 3Postvaccination evaluation of PEDV‐specific IgG by iELISA. (A) Twelve‐week kinetics of PEDV IgG measured by iELISA. (B) Correlation between iELISA‐measured PEDV IgG and neutralizing antibody (NAb) titers.

**Figure:**
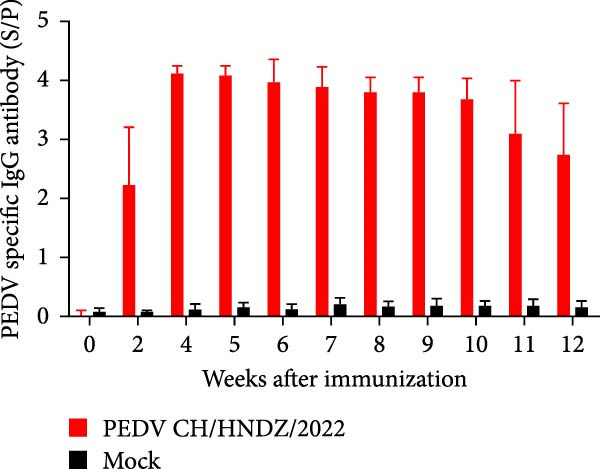
(A)

**Figure:**
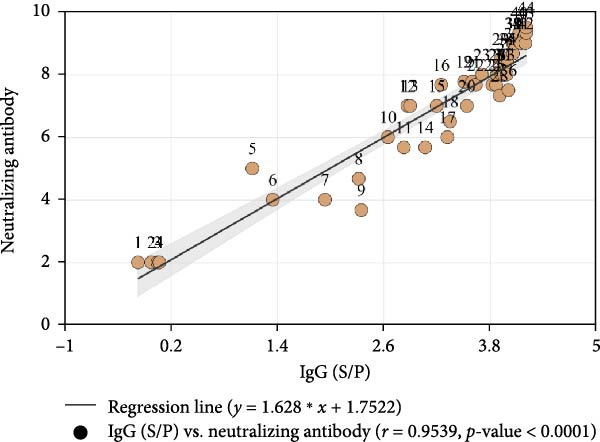
(B)

On a farm undergoing a PEDV outbreak, pregnant sows received two intramuscular doses of a commercial inactivated PED vaccine at 5 and 3 weeks prepartum as per the immunization schedule. Due to ongoing field exposure, piglets became naturally infected with PEDV after birth. Field samples collected during the outbreak were confirmed PEDV‐positive by RT‐qPCR, and sequencing of the S gene revealed that the circulating strain belonged to the G2c genotype. Virus‐neutralizing (VN) antibody titers were determined using the PEDV CH/HNDZ/2022 strain (GenBank accession number PQ316088; G2c genotype), which was also employed for S protein expression in the iELISA assay. The relationship between litter‐level survival at 21 days postinfection and maternal VN and IgG titers measured at farrowing was analyzed, and, within the vaccinated cohort, the association between maternal antibody levels and piglet survival was further examined (Figure 4). Maternal serum VN titers showed the strongest correlation with piglet survival (*r =* 0.89, *p*  < 0.0001; Figure 4A), while maternal anti‐PEDV IgG levels were also highly correlated (*r* = 0.85, *p*  < 0.0001; Figure 4B). These findings demonstrate that the PEDV‐specific IgG iELISA established in this study serves as a reliable surrogate marker of protective immunity, providing a practical tool for field evaluation of vaccine effectiveness and quantification of protective antibody levels in breeding herds.

Figure 4Correlation of maternal antibody levels with piglet survival following sow immunization and piglet PEDV infection. (A) Correlation between sow serum neutralizing antibody (NAb) titers and piglet survival rate. (B) Correlation between sow serum PEDV‐specific IgG levels and piglet survival rate.

**Figure:**
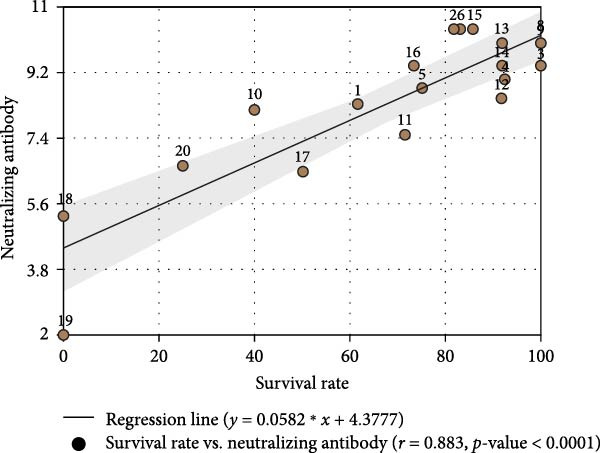
(A)

**Figure:**
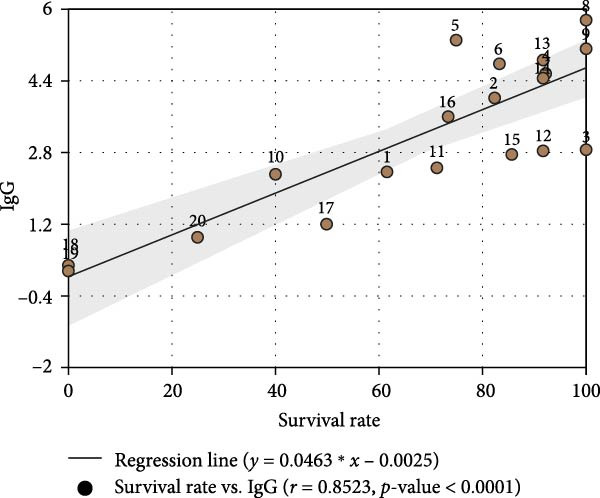
(B)

PEDV is among the most frequently detected and most consequential pathogens affecting swine in field settings [26–28]. Since 2010, highly virulent variant strains (genotype G2) have circulated persistently, undermining the protection conferred by traditional G1 strain‐based vaccines, with frequent vaccine failure particularly in neonatal piglets [29, 30]. Vaccination remains a key strategy to curb PEDV transmission and reduce mortality risk [31]; therefore, longitudinal monitoring of postimmunization antibody levels is warranted [19, 32]. Inactivated vaccines remain widely used in the field to induce protective immunity against PEDV. The correlation between S protein‐specific antibodies and neutralizing antibody (NAb) titers observed in this study is consistent with previous findings that the S protein is the major target of neutralizing antibodies against PEDV [33]. Several studies have demonstrated that antibody responses against the S1 subunit, particularly the S1‐NTD and S1‐RBD domains, are strongly associated with virus neutralization and protection. These findings support the reliability of S protein‐based ELISA assays as effective tools for evaluating immune protection levels in vaccinated herds. Furthermore, the high genetic variability of the PEDV S gene, especially among different genotypes (GIIa, GIIb, GIIc, and S‐INDEL strains), may influence the binding efficiency of antibodies in serological assays. Such sequence divergence can lead to reduced antigen–antibody recognition and potential underestimation of immune responses when using heterologous coating antigens. Therefore, continuous monitoring of circulating PEDV variants and periodic updates of antigen sources are essential to maintain the diagnostic accuracy and epidemiological applicability of S‐based iELISA assays. The spike (S) protein governs receptor binding and tropism and is the dominant target for vaccine development and neutralization [14]. Accordingly, we developed an iELISA using the S protein of the PEDV CH/HNDZ/2022 strain as the coating antigen. To better recapitulate in vivo antigenic conformations, the S protein was recombinantly expressed using a CHO cell system. This platform supports proper folding, assembly, and posttranslational modification to yield near‐native trimers that maximally preserve conformational epitopes—key targets of neutralizing antibodies that are often misrepresented in prokaryotic systems such as *E. coli* [34, 35]. Using CHO‐expressed spike, the iELISA developed here demonstrates high specificity and sensitivity for detecting PEDV‐specific antibodies, with minimal cross‐reactivity to other porcine coronaviruses (e.g., TGEV, PDCoV), thereby reducing false positives [36, 37]. CHO cells also offer favorable process scalability and lot‐to‐lot consistency, enabling large‐scale, standardized antigen manufacture and supporting kit commercialization [38, 39].

We monitored antibody responses following immunization with the inactivated PEDV CH/HNDZ/2022 vaccine and observed a significant correlation between IgG and VN antibodies (Figure 3B). After deploying this vaccine in a herd, analysis of piglet survival following PEDV infection showed that both IgG and NAb levels in piglets were significantly associated with survival. These findings further indicate that monitoring maternal serum IgG can predict the protective outcome of piglets against PEDV, underscoring its value for herd health management. Finally, we established an iELISA for detecting PEDV‐specific IgG in porcine sera, enabling assessment of protective immunity after inactivated vaccination and providing a robust tool for vaccine evaluation.

## 3. Materials and Methods

### 3.1. Reference Sera and Test Kits

Heterologous positive sera against PRRSV, TGEV, PoRVA, and PDCoV were obtained from the in‐house repository of our laboratory. ASFV‐positive serum and PEDV‐negative control serum were purchased from the National Institutes for Food and Drug Control (NIFDC, Beijing, China). A commercial PEDV IgG ELISA kit (Guangdong Biaoyun Biotechnology Co., Ltd., China) was used as a reference assay according to the manufacturer’s instructions. The kit employs purified recombinant PEDV S protein as the coating antigen for the detection of PEDV‐specific IgG antibodies in serum samples.

### 3.2. Virus Strain and Cell Culture

PEDV strain CH/HNDZ/2022 (GenBank accession PQ316088) was maintained and propagated in Vero cells (ATCC CCL‐81) using DMEM (Gibco, USA) supplemented with 7 μg/mL trypsin (Gibco, USA), 100 U/mL penicillin, and 100 μg/mL streptomycin (Gibco, USA), at 37°C in 5% CO_2_.

### 3.3. Development and Optimization of a CHO‐Expressed Spike‐based iELISA

The spike (S) antigen was derived from the PEDV CH/HNDZ/2022 strain (GenBank accession: PQ316088). The codon‐optimized full‐length S gene was cloned into the pCAGGS vector with a C‐terminal His_6_ tag and a tissue plasminogen activator (tPA) signal peptide for secretion. The recombinant plasmid was transiently transfected into CHO‐S cells cultured in CD‐CHO medium (Gibco, USA) using PEI MAX (Polysciences, USA). At 96 h posttransfection, the culture supernatant was collected and clarified by centrifugation (10,000 × *g*, 15 min). The His‐tagged S protein was purified using Ni^2+^‐NTA affinity chromatography (Cytiva HisTrap HP column) followed by size‐exclusion chromatography on a Superdex 200 Increase 10/300 GL column in PBS buffer (pH 7.4).

The purity and integrity of the recombinant S protein were verified by SDS–PAGE under reducing and nonreducing conditions and confirmed by Western blotting with anti‐His monoclonal antibody. The purified protein showed a single dominant band of ~220 kDa, and densitometric analysis indicated a purity greater than 95%. Endotoxin levels were below 0.1 EU/μg, and the protein concentration was determined using a BCA Protein Assay Kit (Thermo Fisher Scientific).

The purified S protein was used as the coating antigen in the iELISA. HRP‐conjugated rabbit antiporcine IgG secondary antibody was purchased from Invitrogen. Using this antigen for plate coating, we optimized iELISA conditions by checkerboard titration, calculating the positive/negative OD_450_ ratio (P/N) and selecting conditions that maximized P/N. Purified antigen was diluted in 0.1 mol/L carbonate–bicarbonate buffer (pH 9.6) to 0.5, 1.0, 1.5, and 2.0 μg/mL. Plates were coated with 100 μL/well at each concentration and incubated overnight at 4°C. Plates were washed three times with PBST, blocked with 200 μL/well of 2% BSA in PBST at 37°C for 3 h. After three additional PBST washes, plates were air‐dried and sealed in foil pouches until use. Serum samples were diluted in 1% BSA in PBST. Diluted sera (100 μL/well) were incubated at 25°C for 1 h. After three PBST washes, 100 μL/well of HRP–rabbit antiporcine IgG (diluted 1:10,000, 1:30,000, or 1:60,000 in a stabilized HRP antibody diluent; Thermo Fisher, Cat. Number 37552) was added and incubated at 25°C for 1 h. Plates were washed three times with PBST, developed with 100 μL/well TMB at room temperature in the dark for 10 min. Reactions were stopped with 100 μL/well 2 mol/L H_2_SO_4_, and absorbance was immediately read at 450 nm. The coating concentration and serum dilution that yielded the maximal P/N were defined as the optimal conditions. Under these optimal antigen and serum settings, we further optimized blocking reagent/type and duration, as well as the secondary antibody dilution. Blocking reagents (5% skim milk, 2% BSA, or 1% gelatin) were tested at 37°C for 1–4 h, and TMB substrate (Huzhou Yingchuang Biotech) incubation was evaluated at 25°C for 5, 10, 15, or 20 min. Primary (serum) incubation times of 30, 60, 90, or 120 min at 25°C and secondary incubation times of 30, 60, 90, or 120 min at 25°C were compared, with additional fine‐tuning at 30, 45, or 60 min where indicated.

### 3.4. Cutoff Determination

Using the optimized iELISA conditions described above, samples were measured at OD_450_. For each specimen, the S/P ratio was calculated as

S/P=ODsample−ODNC/ODPC−ODNC.

A panel of 50 clinically negative and 30 positive sera was tested under identical conditions. The cutoff was derived from the negative panel by computing the mean (xxx) and SD (sss) of S/P values and defining the diagnostic threshold as x + 3sx + 3sx + 3s. In our dataset, the mean S/P of negatives was 0.385 with an SD of 0.134, yielding a cutoff of 0.794; samples with S/P ≥ 0.794 were considered positive and those with S/P < 0.794 negative. The 30 positive sera were used to verify assay sensitivity and validate the cutoff.

### 3.5. Comparison of Sensitivity and Specificity With a Commercial iELISA Kit

Sensitivity assessment: a commercial PEDV IgG kit and our in‐house kit were used to test the same panel of samples. Eight PEDV IgG–positive sera were selected for serial twofold dilution testing. Briefly, 100 μL of serum was used as the stock and diluted in PBS. Step 1: prepare an initial 1:100 dilution. Mix 10 μL of serum stock with 990 μL of PBS to obtain 1:100. From the 1:100 dilution, prepare a 1:600 dilution by adding 100 to 500 μL PBS and vortex thoroughly. As required, further prepare 1:1200, 1:2400, and 1:4800 dilutions from the 1:600 dilution. For example, mixing 50 μL of the 1:600 serum with 50 μL PBS yields a 1:1200 dilution. Samples were assayed either following the procedure above or according to the manufacturer’s instructions. Specificity assessment: sera positive for PDCoV, TGEV, PoRVA, PRRSV, and ASFV, alongside porcine negative sera, was collected. The in‐house kit was used per instructions to evaluate cross‐reactivity with heterologous positive sera.

### 3.6. Preparation of the PEDV CH/HNDZ/2022 Inactivated Vaccine

PEDV CH/HNDZ/2022 was propagated in Vero cells grown in T‐75 flasks (Corning, USA) to a titer of 10^7^ TCID_50_/mL. Infected cultures underwent three freeze–thaw cycles, and the virus‐containing supernatant was clarified by centrifugation. For chemical inactivation, binary ethylenimine (BEI) was added from a 0.2 molar (M) stock to a final concentration of 2 millimolar (mM), followed by incubation at 30°C for 30 h. The reaction was then quenched by adding sodium thiosulfate to a final concentration of 0.2% (w/v) and incubating at 37°C for 20 h to neutralize residual aziridines. The BEI‐inactivated PEDV bulk was formulated with adjuvant 201 (Sino‐Gene, China) according to the manufacturer’s instructions and stored at 4°C until use.

### 3.7. Experimental Immunization of Piglets

Twenty‐four 4‐week‐old piglets with no prior PEDV infection or vaccination history were randomly assigned to two groups (*n* = 12/group) and housed separately. Each vaccinated piglet received 2 mL of the inactivated PEDV preparation at 10^7^ TCID_50_/mL equivalent per dose via intramuscular injection in a prime–boost schedule on day 0 and day 14. Blood samples were collected from each piglet on weeks 0, 2, 4, 5, 6, 7, 8, 9, 10, 11, and 12 for serological assays (IgG iELISA and virus neutralization, as described elsewhere).

### 3.8. Field Vaccination of Pregnant Sows and Survival Monitoring of Piglets

On a farm experiencing a PEDV outbreak, 20 pregnant sows were immunized intramuscularly with an commercial inactivated PED vaccine on a two‐dose schedule at −5 and −3 weeks before farrowing. Two weeks after the second dose, sow sera were collected for PEDV‐specific IgG iELISA and VN antibody measurements. Piglet survival in each litter was monitored for 21 days after birth to evaluate the association between maternal antibody levels and litter‐level survival outcomes.

### 3.9. Neutralization Assay for PEDV‐Specific Antibodies

Serum samples were heat‐inactivated at 56°C for 30 min and subjected to twofold serial dilutions in DMEM (starting dilution as indicated for each experiment). An equal volume of PEDV suspension containing 200 TCID_50_ per 100 µL—back‐titrated on the day of use—was added to each diluted serum, mixed gently, and incubated at 37°C for 1 h to allow antibody–virus binding. Vero cells (ATCC CCL‐81) were seeded in 96‐well plates 24 h prior to infection to reach ~90% confluence. Monolayers were rinsed once with PBS and inoculated in duplicate with 100 µL of each serum–virus mixture per well. After adsorption for 90 min at 37°C (5% CO_2_) with gentle rocking every 15 min, the inoculum was removed, cells were washed twice with PBS, and 100–150 µL/well of maintenance medium (DMEM supplemented with TPCK‐treated trypsin [L‐(tosylamido‐2‐phenyl) ethyl chloromethyl ketone‐treated trypsin] at 10 µg/mL; serum‐free or ≤2% FBS as optimized) was added. Plates were incubated for 48 h at 37°C. Wells were examined daily by light microscopy for PEDV‐specific cytopathic effects (CPE). Each plate included a cell‐only control, a virus‐only control, and a reference positive serum. NAb titers were expressed as the reciprocal of the highest serum dilution that prevented CPE in ≥50% of replicate wells relative to the virus control (NT_50_). When required, NT_50_ values were calculated by the Reed–Muench method.

### 3.10. Correlation Analysis

At the litter level, we assessed the association between maternal antibody levels and piglet survival (%). For each litter, survival was calculated as the number of animals alive at 21 dpi (days postimmunization) or at the humane endpoint, whichever occurred first, divided by the number enrolled × 100. Sow serum NAb titers were measured by virus neutralization test (VNT); endpoint titers were determined by twofold serial dilution and log_2_‐transformed for analysis. IgG antibodies were quantified by iELISA as described herein; each sample was tested in ≥2 intraplate technical replicates, the mean OD was used to derive S/P values or endpoint titers. Correlation analysis. For each sample, the first analyte was placed on the x‐axis and the second analyte on the y‐axis. Two‐sided Pearson correlation was computed (reporting *r*, 95% confidence interval (CI), and *p*), and an ordinary least squares (OLS) model (*y* = *β*_0_ + *β*_1_ *x*) was fitted, reporting the slope with its 95% CI. Log_2_/log_10_ transformation was applied when needed to improve linearity. Scatterplots were color coded by group with an overlaid regression line (95% CI); the top‐left annotation displayed the fitted equation and R ( =*r*) with *p*. Analyses were performed in R 4.3+ or Python 3.10+, with *α* = 0.05 (two‐sided).

### 3.11. Statistical Analysis

Statistical analyses were performed in GraphPad Prism 8. Unless stated otherwise, data are presented as mean ± SD with individual data points. Two‐group comparisons used two‐tailed unpaired *t*‐tests; when assumptions were violated (Shapiro–Wilk, Brown–Forsythe), the Mann–Whitney test was applied. Multigroup data were analyzed by one‐way ANOVA with Holm–Šídák correction. Statistical significance was set at *p*  < 0.05; significance is denoted as  ^∗^ *p*  < 0.05,  ^∗∗^ *p*  < 0.01,  ^∗∗∗^ *p*  < 0.001,  ^∗∗∗∗^ *p*  < 0.0001.

## Ethics Statement

Animal ethics approval: All animal procedures involving piglets were reviewed and approved by the Animal Ethics Committee of Guangdong Ocean University, China (Approval Number: GDOU‐CCAS‐2024‐027). All procedures complied with national and institutional guidelines for the care and use of laboratory animals and adhered to the ARRIVE reporting recommendations where applicable.

## Conflicts of Interest

The authors declare no conflicts of interest.

## Author Contributions

Guangming Ma provided the original data and conducted experimental data collection. Guangli Hu was responsible for writing and reviewing the manuscript. Guangcai Ren supplied the experimental materials. Wang Ma contributed to the experimental design. Shuqiong Zhang provided the experimental platform and reviewed the manuscript. Hanqin Shen supported the animal experiments. Yuanjia Liu designed the overall study, supervised data organization, and reviewed the manuscript.

## Funding

This research was supported by the Start‐up Fund for Scientific Research at Guangdong Ocean University (Grant No. 060302052301).

## Data Availability

The data that support the findings of this study are available from the corresponding author upon reasonable request.
